# Identification of novel prognostic biomarkers for thyroid cancer by integrated transcriptome analysis of metastasis-associated genes

**DOI:** 10.3389/fonc.2025.1536270

**Published:** 2025-05-19

**Authors:** Bushra Alnwisser, Salman Alshehri, Amal Qattan, Minjing Zou, Abdelilah Aboussekhra, Ali S. Alzahrani, Yufei Shi

**Affiliations:** ^1^ Department of Molecular Oncology, King Faisal Specialist Hospital and Research Centre, Riyadh, Saudi Arabia; ^2^ King Abdulaziz and His Companions Foundation for Giftedness and Creativity, Riyadh, Saudi Arabia; ^3^ Department of Medicine, King Faisal Specialist Hospital and Research Centre, Riyadh, Saudi Arabia

**Keywords:** metastasis-associated gene, prognosis, biomarker, metastasis, thyroid cancer

## Abstract

**Introduction:**

Distant metastasis (DM) is the most important prognostic factor affecting the overall survival (OS) of thyroid cancer. The current study aimed to discover prognostic biomarkers to predict thyroid cancer survival, particularly papillary thyroid carcinoma (PTC), the most common subtype of thyroid cancer.

**Methods:**

Four RNA sequencing (RNA-Seq) datasets of experimental lung metastasis from four transgenic mouse models of PTC, follicular thyroid cancer (FTC), poorly differentiated thyroid cancer (PDTC), and anaplastic thyroid cancer (ATC) were integrated to screen for candidate genes involved in DM. The Cancer Genome Atlas-Thyroid Cancer (TCGA-THCA) dataset was used to validate the candidate genes.

**Results:**

A total of 105 upregulated and 25 downregulated differentially expressed genes (DEGs) were identified to be present in all four datasets. Regulation of cytokine production, inflammation, immune checkpoint regulation, and MAPK/ERK cascade were major enriched pathways in metastatic tumor cells. Seven genes were identified whose overexpression was present in 63 of 498 PTC patients (13%) and was associated with poor OS (p < 0.01). Clinically, the seven-gene expression signature was associated with older age at the diagnosis, late stage of tumor, tall cell variant, and higher aneuploidy and hypoxia score. Mutation load was increased in patients with seven-gene expression signature: 26 samples had more than one driver mutation (47%, 26/55). Deep deletions in other chromosomal loci were frequently found in patients with BRAFV600E mutations. In contrast, only 7% of patients without a seven-gene expression signature had more than one driver mutation (24/243). Increased chromosomal instability was also observed in patients with a seven gene expression signature.

**Conclusion:**

The seven-gene expression signature is associated with poor prognosis and chromosomal instability. These genes may be useful biomarkers for risk stratification for DM and help decision-making in initial surgical recommendations.

## Introduction

Thyroid cancer is the most common malignancy in the endocrine system and is commonly classified into papillary thyroid carcinoma (PTC), follicular thyroid cancer (FTC), poorly differentiated thyroid cancer (PDTC), and anaplastic thyroid cancer (ATC) based on the histological type. PTC is the most predominant type of thyroid cancer, accounting for more than 85% of the cases followed by FTC (5%–10%), PDTC (4%–7%), and ATC (approximately 2%) (1, 2). PTC and FTC have excellent prognoses with a 10-year survival of up to 90%; PDTC has poorer prognosis with a 5-year survival at 66%; ATC is highly virulent with a mean survival of less than 8 months (2–4).

The main drivers in thyroid cancer pathogenesis are single-point mutations and gene fusions in components of MAPK and PI3K/Akt pathways such as point mutations of *BRAF*, *RAS*, *PIK3CA*, and *AKT1* or gene fusions of *BRAF*, *RET*, *ALK*, and *NTRK*. Other important genetic alterations in the more advanced types of thyroid cancer include mutations in the *TERT* promoter, *EIF1AX*, *MED12*, *RBM10*, *CTNNB1*, and *TP53* (5, 6). The *BRAF* ^V600E^ mutation is the most frequent genetic alteration in PTC with an overall rate of 60% (7).

Metastasis is the leading cause of thyroid cancer mortality and morbidity (8, 9). Distant metastasis (DM) occurs in approximately 10% of PTC and up to 25% of FTC patients. The lungs (~80%) and bones (~25%) are the most common sites for distant metastasis with overall survival (OS) of approximately 10 months in patients with metastases to the lung and 23 months in patients with metastases to the bone (10–13). Early identification and proper treatment of these patients would improve their OS. Recently, molecular testing has been used for risk stratification for DM in patients with differentiated thyroid cancer (PTC and FTC) (14). The study found that most patients with DM had late‐hit mutations in *TERT*, *TP53*, or *PIK3CA*. Gene expression signature for risk stratification has not been investigated in patients with PTC. There may be other molecular markers yet to be discovered for risk stratification for DM in patients without *TERT*, *TP53*, or *PIK3CA* mutations.

In the present study, we characterized the gene expression profile associated with thyroid cancer metastasis using cell lines derived from genetically engineered mouse models of PTC, FTC, PDTC, and ATC. We identified seven genes whose overexpression was associated with poor OS.

## Materials and methods

### Experimental animals

Athymic BALB/c-nu/nu (nude mice) were acquired from Jackson Laboratory. Mice were provided with autoclaved food and water *ad libitum*. The study was approved by the Animal Care and Use Committee of the institution and was conducted in compliance with the Public Health Service Guidelines for the Care and Use of Animals in Research.

### Thyroid cancer cell lines

Four murine thyroid cancer cell lines derived from genetically engineered mouse models of PTC, FTC, PDTC, and ATC were established from primary tumors: PTC with *Braf* ^V600E^ mutation (PTC), FTC with *Kras* ^G12D^ mutation (FTC), PDTC with both *Kras^G12D^* and *Cdkn2a^null^* mutations (PDTC), and ATC with both *Braf* ^V600E^ and *Trp53* ^null^ mutations (ATC). The establishment of PTC, FTC, and ATC strains was described previously (15–18). PDTC strain was established by cross-breeding among *Kras^G12D^*, TPO-Cre, and *Cdkn2a^null^* (strain 01XE4 obtained from The NCI Mouse Repository, https://frederick.cancer.gov/Resources/Repositories/nci-mouse-repository/MouseModels/AvailableStrains#/StrainDetails/79). PDTC was developed from a 13-month-old mouse with both *Kras^G12D^* and *Cdkn2a^null^* mutations. The PDTC cell line was established from the tumor. Thyroid origin was confirmed by genotyping. The cell lines were maintained in DMEM/F12 growth medium containing 10% fetal bovine serum, 100 units/mL penicillin, and 100 μg/mL streptomycin.

### Metastatic thyroid cancer cell lines

**Figure 1:**
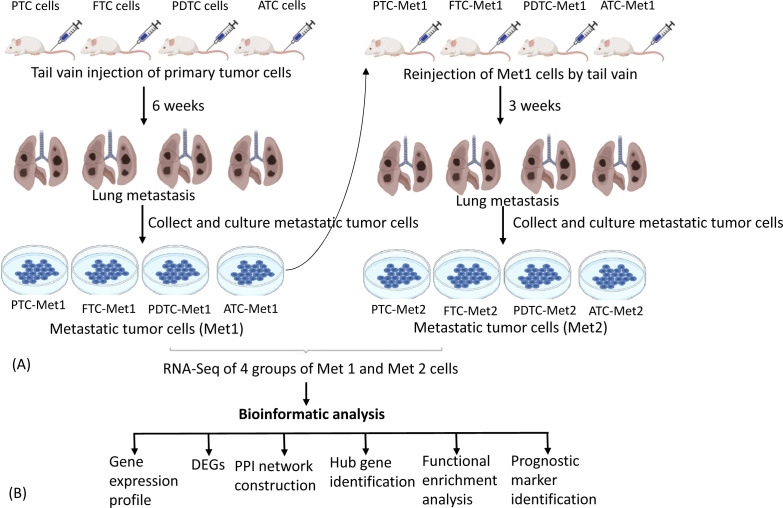
Experimental lung metastasis. **(A)** Schematic diagram summarizing experimental lung metastasis procedure. PTC-Prim, FTC-Prim, PDTC-Prim, or ATC-Prim cells (1 × 10^6^ cells) were injected into the tail vein of five nude mice for each group. Six weeks after injection, lung metastatic tumors were collected to establish Met1 cell lines. The established Met1 cells (PTC-Met1, FTC-Met1, PDTC-Met1, or ATC-Met1) were re-injected (1 × 10^6^ cells) to a new group of nude mice (n = 5 for each group) via tail vein for enrichment of cells with high metastatic potential. Lung metastatic tumors were harvested 3 weeks following injection to establish Met2 cell lines (PTC-Met2, FTC-Met2, PDTC-Met2, or ATC-Met2). Primary, Met1, and Met2 tumor cells were subject to RNA sequencing to identify differentially expressed genes (DEGs). **(B)** Flowchart showing the workflow for the integrated bioinformatic analysis DEGs. PTC, papillary thyroid carcinoma; FTC, follicular thyroid cancer; PDTC, poorly differentiated thyroid cancer; ATC, anaplastic thyroid cancer.

To establish pulmonary metastatic thyroid cancer cell lines, 1 × 10^6^ PTC, FTC, PDTC, or ATC cells were injected into the tail vein of five nude mice for each group. Six weeks after injection, pulmonary metastatic tumors were collected aseptically from the mice using blunt dissection, then mechanically dissociated by mincing and passing through a 40-μM mesh sterile screen, and suspended in DMEM/F12 growth medium for 3 months with a total of six passages to eliminate contaminated stromal fibroblasts, lymphocytes, and microphages present in the tumor cell culture. The primary cells were considered permanent cell lines after six passages. The established metastatic cell lines were named PTC-Met1, FTC-Met1, PDTC-Met1, and ATC-Met1. They were re-injected (1 × 10^6^ cells) to a new group of nude mice (n = 5 for each group) via tail vein for enrichment of cells with high metastatic potential. Three weeks following injection, lung metastatic tumors were harvested and propagated in DMEM/Ham’s F12 growth medium for 3 months with at least six passages. The metastatic cell lines were named PTC-Met2, FTC-Met2, PDTC-Met2, and ATC-Met2. The experimental procedures are summarized in **Figure 1**.

### RNA sequencing analysis

RNA sequencing (RNA-Seq) was used for quantification of differentially expressed genes (DEGs) between primary (PTC, FTC, PDTC, or ATC) and metastatic thyroid cancer cell lines: PTC-Met1, FTC-Met1, PDTC-Met1, and ATC-Met1, or PTC-Met2, FTC-Met2, PDTC-Met2, and ATC-Met2 cell lines. Total RNA from cell lines was isolated, and libraries were constructed using an Illumina (San Diego, CA, USA) TruSeq RNA Library Prep kit according to the manufacturer’s procedure. Sequencing was performed on Illumina HiSeq 4000 with at least 20 million clean reads. The significant DEGs were selected based on the following criteria: Log2 fold change >2, false discovery rate (FDR) <0.001, and p-value from difference test <0.01. Gene list annotation and enrichment of biological pathways were performed using Metascape (https://metascape.org/gp/index.html#/main/step1).

### The protein–protein interaction network construction and hub gene identification

The common DEGs were analyzed using the Search Tool for the Retrieval of Interacting Genes (STRING; ver.12.0, https://string-db.org/) database for the construction of a protein–protein interaction (PPI) network with a confidence score of ≥0.4. The PPI network was then analyzed and visualized using Cytoscape software (ver.3.10.2, https://cytoscape.org). For the hub gene identification, CytoHubba, a plug-in of Cytoscape, was used. Genes with the degree of a node >10 (with more than 10 interacting genes) were considered hub genes.

### Identification of genes associated with poor prognosis

The association of metastasis-related genes with OS was performed by the Kaplan–Meier analysis using TCGA-THCA mRNA expression dataset (n = 498) and cBioPortal For Cancer Genomics (https://www.cbioportal.org/).

### Statistical analysis

Prism was used in statistical analysis. Chi-squared test was used when the data were not normally distributed, and the *t*-test was used when the data were normally distributed. p-Value <0.05 was considered significant. Time-to-event endpoints were visualized using the Kaplan–Meier curves, and differences in OS were compared using Cox’s proportional hazards model.

## Results

### Identification of common DEGs

**Figure 2:**
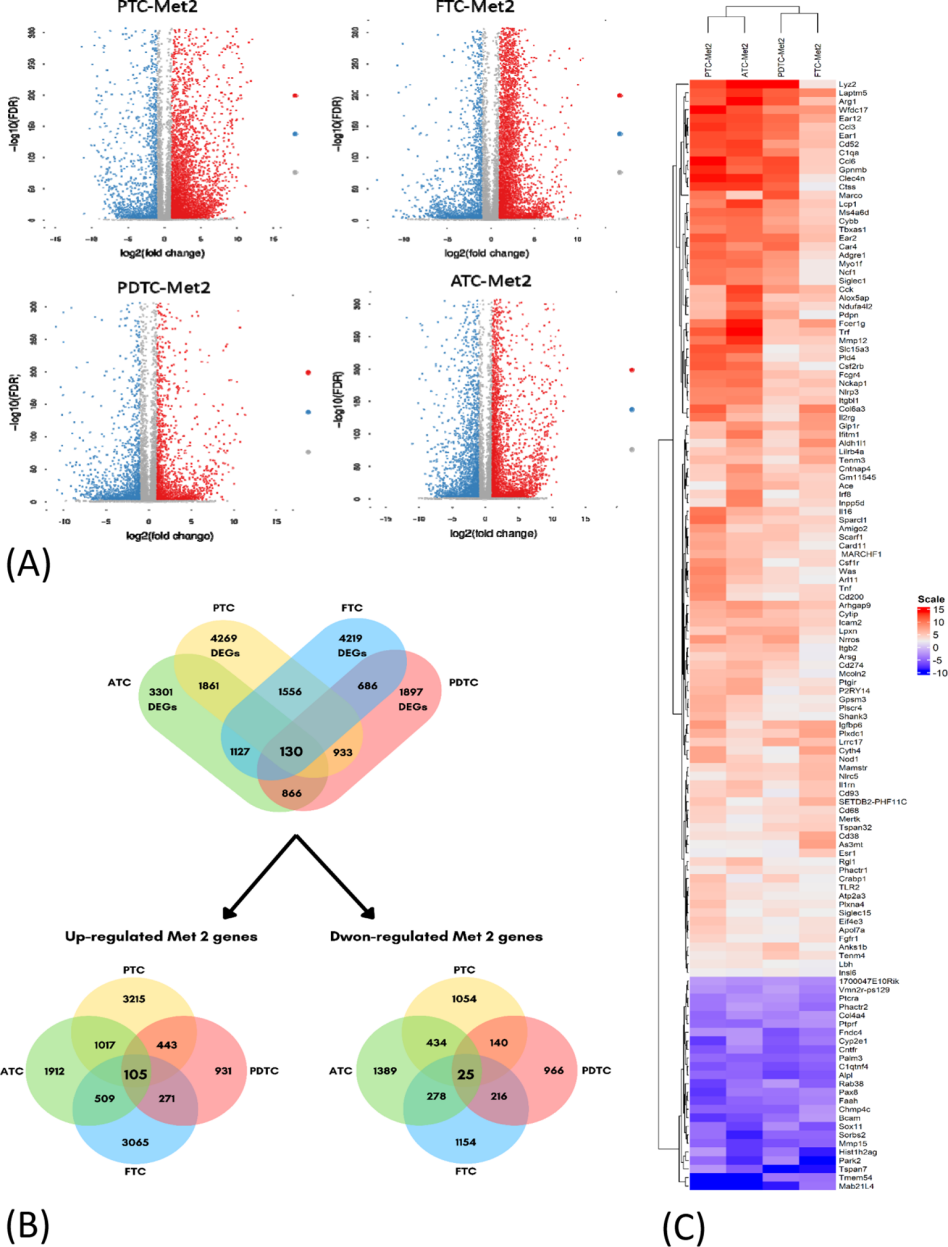
Identification of differentially expressed genes (DEGs) in four datasets of thyroid cancer cell lines. **(A)** Volcano plots of DEGs in the four indicated datasets (PTC-Met2, FTC-Met2, PDTC-Met2, and ATC-Met2). Genes with false discovery rate (FDR) < 0.05 and FC > 1.0 or < −1.0 are considered DEGs for each dataset. Red, upregulated genes; blue, downregulated genes; gray, no DEGs. **(B)** The Venn diagram of DEGs showing the intersection of PTC-Met2, FTC-Met2, PDTC-Met2, and ATC-Met2 cell lines. A total of 130 DEGs are present in all four cell lines including 105 up- and 25 downregulated DEGS. **(C)** Heatmaps of common DEGs. Log2 mRNA intensities were scaled and clustered using hierarchical clustering. PTC, papillary thyroid carcinoma; FTC, follicular thyroid cancer; PDTC, poorly differentiated thyroid cancer; ATC, anaplastic thyroid cancer.

Significant DEGs were identified from RNA-Seq results. A total of 4,269 DEGs were found in the PTC (PTC *vs*. PTC-Met2), 4,219 DEGs in FTC (FTC *vs*. FTC-Met2), 1,897 in PDTC (PDTC *vs*. PDTC-Met2), and 3,301 in ATC (ATC *vs*. ATC-Met2) datasets (**Figure 2A**). A total of 130 common DEGs were found after the integration of four datasets, including 105 upregulated and 25 downregulated genes (Log2 fold change >2) present in all four Met2 cell lines (**Figure 2B**; **Supplementary Table 1**). The expression levels of most DEGs were increased in Met2 as compared to Met1 cells. Many DEGs not detected in Met1 cells also appeared in Met2 cells (**Supplementary Table 1**). These data indicate the enrichment of DEGs in Met2 cells and suggest that these DEGs may be involved in DM. For example, “don’t eat me” signal expression was either no change or mildly increased in Met1 cells but significantly increased in Met2 cells such as Cd274 (PD-L1), Cd52, and Tbxas1 (**Table 1**), indicating that these genes may play a significant role in immune evasion of metastatic cancer cells. Strictly speaking, Tbxas1 is not a “don’t eat me’ signal”, but it indirectly helps cancer cells evade immune elimination via platelet activation and aggregation (19). Met2 cells also took 3 weeks less than Met1 cells (3 *vs*. 6 weeks) to form lung metastasis, indicating enrichment of tumor cells with high metastatic potential.

**Table 1:** “Don’t eat me” signal expression in Met1 and Met 2 cells.

Gene symbol	FTC-Met1*	PDTC-Met1*	PTC-Met1*	ATC-Met1*	FTC-Met2*	PDTC-Met2*	PTC-Met2*	ATC-Met2*
Cd274	−0.44	2.8	0.58	3.59	**2.62**	**4.39**	**5.08**	**6.45**
Cd47	1.66	0.07	0.9	0.28	2.14	0.62	2.24	0.46
B2m (beta-2-microglobulin)	1.4	0.03	2.07	−1.18	2.1	1.2	3.6	−0.36
Cd24	−0.37	−1.9	−6.02	−3.8	−0.41	−5	−4	−1.53
Cd52	4.25	1.85	ND	3.17	**5.7**	**7.7**	**12**	**12.76**
Tbxas1 (thromboxane-A synthase 1)	ND	3.91	ND	3.7	**5.36**	**7.89**	**9.2**	**10.33**

Significant enrichment of Cd274 (PD-L1), Cd52, and Tbxas1 expression is highlighted in bold.

ND, not detected; DEGs, differentially expressed genes; FTC, follicular thyroid cancer; PDTC, poorly differentiated thyroid cancer; PTC, papillary thyroid carcinoma; ATC, anaplastic thyroid cancer.

*Log2 fold change *vs*. control of DEGs.

### Protein–protein interaction network construction and biological function analysis

**Figure 3:**
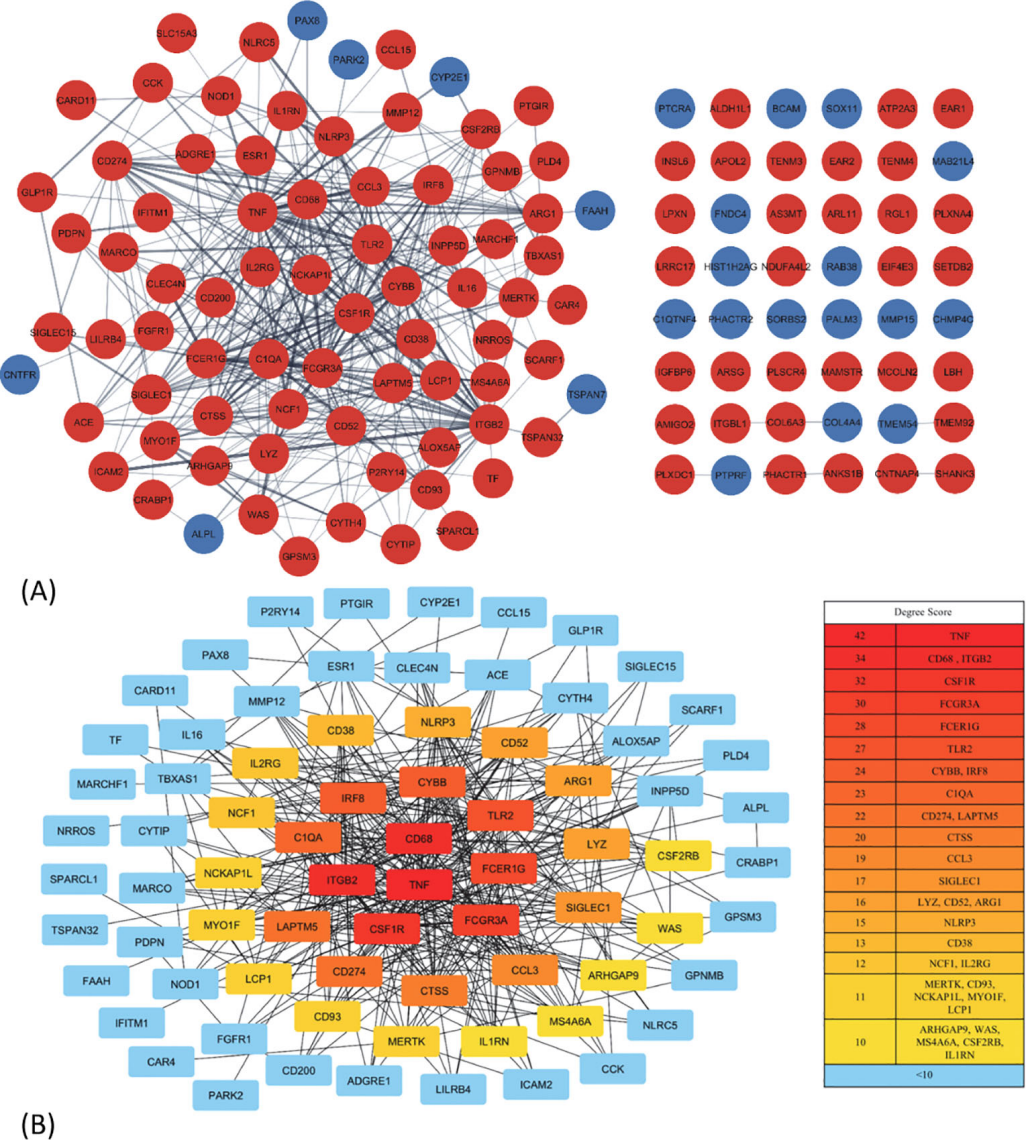
The protein–protein interaction (PPI) network construction and hub gene identification. **(A)** The PPI network of 130 common DEGs. The network was constructed by STRING and analyzed using Cytoscape. Some of them (48 genes) were not able to be incorporated into the PPI network. The red and blue nodes represent the up- and downregulated genes, respectively. **(B)** The interaction and ranking of 32 hub genes. The hub genes were visualized using the degree algorithm of the CytoHubba plugin. The color gradient from red to yellow corresponds to the degree score, with red indicating higher scores and yellow indicating lower scores; the genes with <10 connections are represented by blue. DEGs, differentially expressed genes.

**Figure 4:**
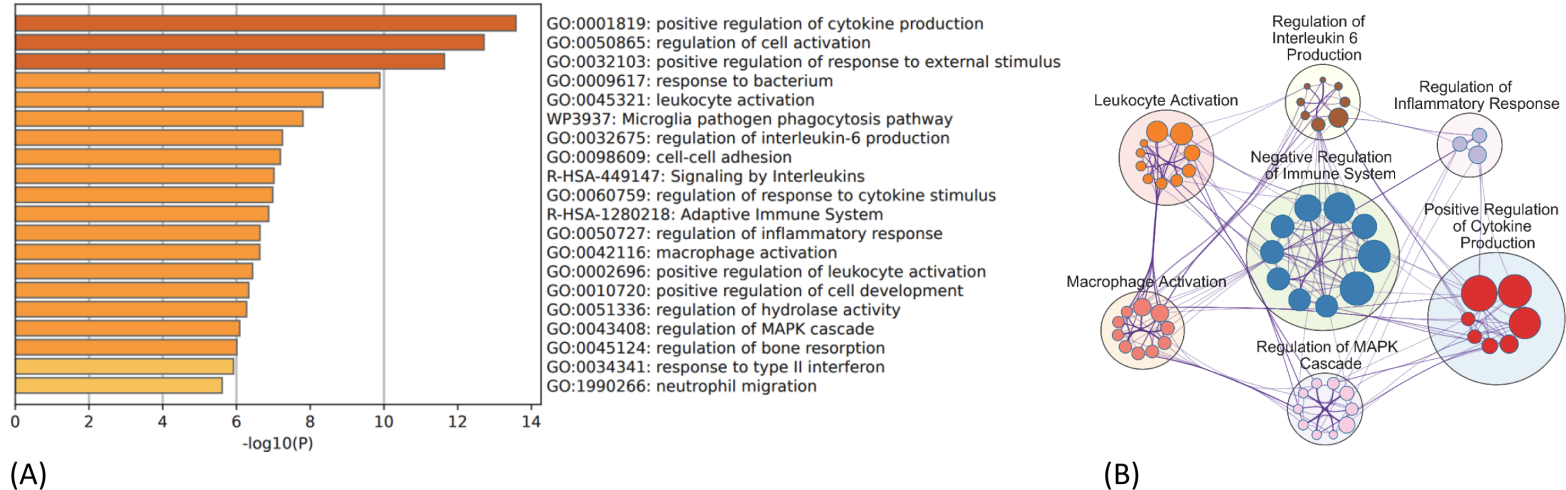
GO and KEGG pathway enrichment analysis of 130 common DEGs. **(A)** Top 20 enriched ontology clusters of DEGs are shown. **(B)** Visualization of meta-analysis results of key enriched ontology clusters of DEGs. The network shows significant interactions among key signaling pathways involved in thyroid cancer metastasis such as regulation of cytokine production, inflammation, immune checkpoint regulation, MAPK/ERK cascade, and leukocyte/macrophage activation process. The big circle represents main gene cluster. The small node indicates each biological process within the main gene cluster and is ranked by logP value (bigger node indicates smaller p-value). The gene list and their involvement in multiple signaling pathways are shown in **Supplementary Table 3**. GO, Gene Ontology; KEGG, Kyoto Encyclopedia of Genes and Genomes; DEGs, differentially expressed genes.

The PPI network for the 130 DEGs was constructed after they were imported to STRING (**Figure 3A**). According to the ranking generated by the Degree algorithm, 32 hub genes were identified (**Figure 3B**). Their full names and related functions are shown in **Supplementary Table 2**. Many of them have been reported to be involved in metastasis in different cancer types. Gene ontology and pathway enrichment analysis revealed the top 20 gene clusters in the regulation of cytokine production, inflammatory and negative regulation of immune response, neutrophil migration and phagocytosis, and MAPK cascade (**Figure 4A**). Visualization of these gene clusters by the PPI network demonstrated significant cross interactions among different pathways (**Figure 4B**). The gene list of enriched clusters and their involvement in multiple signaling pathways are shown in **Supplementary Table 3**.

### Identification of gene expression signature associated with poor survival

**Figure 5:**
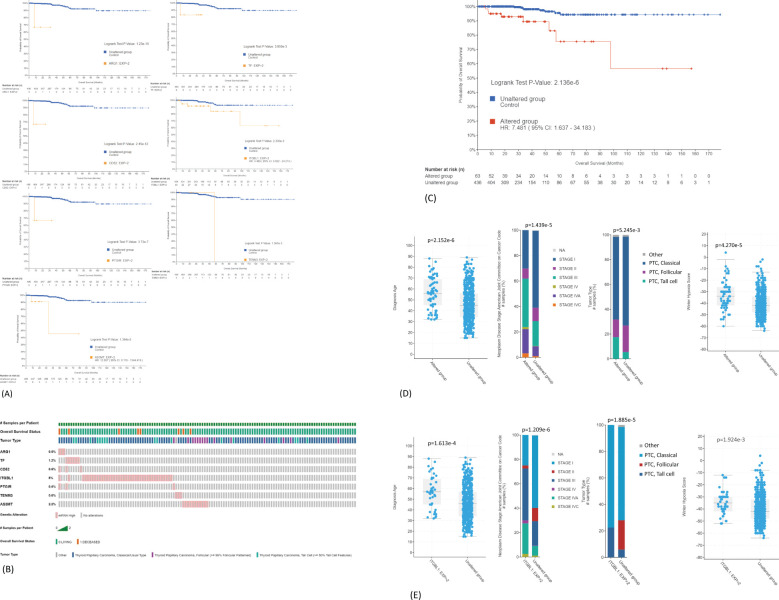
Prediction of DEGs on OS of PTC patients. **(A)** Association of ARG1, TF, CD52, ITGBL1, PTGIR, TENM3, or AS3MT overexpression with poor OS. TCGA-THCA mRNA dataset (n = 498) was used for Kaplan–Meier analyses: mRNA expression z-scores relative to normal samples (log RNA Seq V2 RSEM). **(B)** Distribution of seven-gene expression signature among PTC samples. Overexpression is present in 63 samples (13%, 63/498). **(C)** Association of seven-gene expression signature as a group with poor OS. **(D)** Correlation of seven-gene expression signature with clinical features of PTC. The overexpression is significantly associated with old age at diagnosis, late disease stage, tall cell variant, and higher hypoxia score. **(E)** Correlation of ITGBL1 overexpression with clinical features of PTC. DEGs, differentially expressed genes; OS, overall survival; PTC, papillary thyroid carcinoma.

The association of common DEGs with OS of PTC patients was analyzed using TCGA-THCA dataset (n = 498). Seven genes were identified, and their overexpression was associated with poor OS (ARG1, TF, CD52, ITGBL1, PTGIR, TENM3, and AS3MT) (**Figure 5A**). The distribution of seven-gene expression signature among PTC samples is shown in **Figure 5B**. As shown in **Table 2**, the expression of this group of genes was higher in Met2 cells than in Met1 cells, indicating that they may participate in DM. Since most of these genes were overexpressed in less than 2% of samples except for ITGBL1 (**Figure 5B**), we analyzed them as a group (13% samples, 63/498) for prognosis prediction and clinical attributes. The seven-gene expression signature was significantly associated with poor OS (p < 0.0001, **Figure 5C**). The patients with seven-gene expression signature were associated with old age at diagnosis [56 *vs*. 45 years of median age (p < 0.0001)], late disease stage [38% (24/63) *vs*. 20% (85/435) in stage III and 24% (15/63) *vs*. 9% (39/435) in stage IV tumors (p < 0.0001)], tall cell variant [18% (11/63) *vs*. 6% (24/435) (p < 0.01)], and higher hypoxia score [−34 *vs*.− 38 of medium (p < 0.0001)] (**Figure 5D**). The overexpression of ITGBL1 was found in 8% of samples (40/498). Its overexpression was also associated with old age at diagnosis [7 *vs*. 46 years of median age (p < 0.0001)], late disease stage [43% (17/40) *vs*. 20% (92/458) in stage III and 30% (12/40) *vs*. 9% (42/458) in stage IV tumors (p < 0.0001)], tall cell variant [23% (9/40) *vs*. 6% (26/458) (p < 0.0001)], and higher hypoxia score [−35 *vs*. −42 of median value (p < 0.01)] (**Figure 5E**). Thus, ITGBL1 overexpression may be the most useful single gene marker for poor prognosis prediction.

**Table 2:** Differential expression of seven prognostic marker genes in Met1 and Met 2 cells.

Gene	FTC-Met1*	FTC-Met2*	PDTC-Met1*	PDTC-Met2*	PTC-Met1*	PTC-Met2*	ATC-Met1*	ATC-Met2*
Arg1 (arginase 1)	8.11	5.39	6.63	11.56	1.58	11.22	5.09	15.65
As3mt (arsenite methyltransferase)	6.08	7.5	1.91	2.16	2.30	2.45	−0.49	2.60
Cd52	4.25	5.7	1.85	7.71	ND	12.10	ND	12.76
Itgbl1 (integrin subunit beta-like 1)	2.75	5.11	3.86	4.16	7.88	8.87	9.38	9.19
Ptgir (prostaglandin I2 receptor)	2.32	3.46	1.30	3.16	1.38	5.68	ND	7.04
Tenm3 (teneurin transmembrane protein 3)	4.05	7.16	3	3	6.27	5.81	2.12	5.57
Trf (transferrin)	9.66	5.9	1.61	5.07	2.32	11.59	3.0	13.99

ND, not detected; FTC, follicular thyroid cancer; PDTC, poorly differentiated thyroid cancer; PTC, papillary thyroid carcinoma; ATC, anaplastic thyroid cancer; DEGs, differentially expressed genes.

*Log2 fold change *vs*. control of DEGs.

### Mutation load in samples with seven-gene expression signature

**Figure 6:**
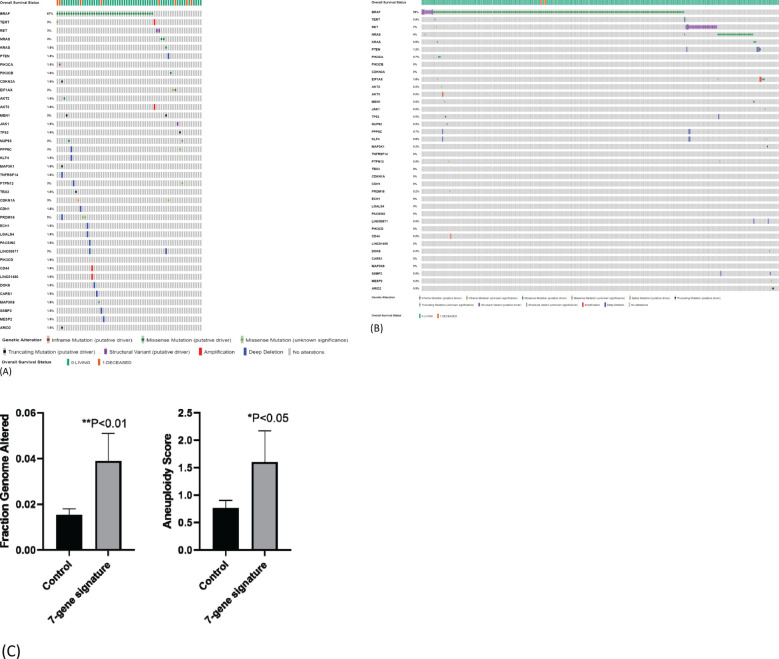
Mutation load in samples with seven-gene expression signature. **(A)** Mutational load in samples with seven-gene expression signature. TCGA-THCA data were used for analysis. Higher frequency of gene mutations is observed in 55 PTC samples with seven-gene expression signature. **(B)** Mutational load in samples without seven-gene expression signature. Lower frequency of gene mutations in 343 PTC samples without seven-gene expression signature. **(C)** Fraction genome altered and aneuploidy score in patients with or without seven-gene expression signature. PTC, papillary thyroid carcinoma.

We next compared mutational load between samples with or without seven-gene expression signature using TCGA-THCA dataset. We chose 38 genes whose mutations have been reported to be involved in carcinogenesis including genes known to be driver mutations in thyroid carcinogenesis. Among samples with seven-gene expression signature, 26 samples had more than one driver mutation (47%, 26/55) (**Figure 6A**). Deep homozygous deletions were frequently found in samples with *BRAF* ^V600E^ mutation (**Figure 6A**). Among patients without seven-gene expression signature, only 24 patients had more than one driver mutations (7%, 24/343) (**Figure 6B**). The difference is highly statistically significant (p < 0.00001). These data suggest that the seven-gene expression signature may reflect underlying increased chromosomal instability. Indeed, as shown in **Figure 6C**, patients with seven-gene expression signature had a higher rate of fraction of genome altered (FGA; the percentage of genome that has been affected by copy number gains or losses) than those without: 0.0399 ± 0.012 (mean ± SEM, n = 62) *vs.* 0.0153 ± 0.003 (n = 434, p < 0.01). Aneuploidy score was also increased in patients with seven-gene expression signature than those without: 1.60 ± 0.57 (mean ± SEM, n = 60) *vs.* 0.77 ± 0.14 (n = 405). These genetic changes may drive the metastatic transformation of tumor cells, resulting in distant metastasis.

## Discussion

The main purpose of the study was to identify common DEGs involved in DM of thyroid cancer for prognosis prediction and revelation of potential mechanisms. We used four cell lines derived from four different thyroid cancer transgenic mouse models ranging from well-differentiated (PTC and FTC) to PDTC and ATC. This would help us find common DEGs relevant to all four tumor types. We validated the common DEGs using TCGA-THCA dataset and identified a seven-gene expression signature whose overexpression was associated with poor OS of PTC patients.

Hub gene identification is commonly used as an initial step in searching for prognostic biomarkers in many studies (20–22). While this approach is valuable, it has limitations, as not all candidate genes may be identified as hub genes. In the current study, among the seven candidate genes for prognostic prediction (ARG1, CD52, ITGBL1, TF, PTGIR, TENM3, and AS3MT), only ARG1 and CD52 were identified as hub genes. For instance, ITGBL1, an integrin subunit beta-like 1 protein, was not classified as a hub gene, but its overexpression was significantly associated with poor overall survival and advanced stages of thyroid cancer. Functionally, ITGBL1 promotes metastatic tumor growth by fostering a fibroblast niche through the secretion of pro-inflammatory cytokines such as IL-6 and IL-8 (23). Therefore, expanding the search beyond hub genes may help identify additional candidate genes that could serve as valuable biomarkers.

Thyroid cancer patients typically have high rates of early diagnosis and treatment. As a result, most patients do not develop distant metastases. Therefore, the current findings may be more relevant for predicting high-risk lymph node metastasis and guiding surgical lymph node removal. Notably, the seven-gene expression signature is significantly associated with advanced stages of thyroid cancer, particularly the tall cell variant, which is often linked to lymph node metastasis.

We have also explored potential pathways driving thyroid cancer metastasis. Gene Ontology (GO) and Kyoto Encyclopedia of Genes and Genomes (KEGG) pathway enrichment analyses of common DEGs revealed significant enrichment in pathways related to cytokine production, negative regulation of immune responses, neutrophil migration, phagocytosis, cell–cell adhesion, and MAPK signaling. Many of these pathways have been implicated in thyroid cancer progression (24, 25). Our findings suggest that inflammation, immune checkpoint regulation, and platelet aggregation play critical roles in thyroid cancer metastasis. Elevated expression of inflammatory cytokines/chemokines such as IL-6, Ccl3, Irf8, and Tnf was observed in Met2 cells, with Ccl3, Irf8, and Tnf identified as hub genes. Notably, a significant proportion of these hub genes are involved in endoplasmic reticulum (ER) stress and inflammation (Ccl3, Irf8, Tnf, Cybb, Nlrp3, Ncf1, Il2rg, Il1rn, and Tlr2) or immune checkpoint regulation (Cd274, Cd52, Cd38, Arg1, Csf2rb, and Fcer1g). Inflammation, typically a host response to pathogens, can be triggered by endogenous mediators (cytokines/chemokines) through tall-like receptors like Tlr2 to promote tumor progression and metastasis (26). Inhibition of Tlr2 has been shown to reduce pulmonary metastases in melanoma (27). Elevated expression of Cd274 (PD-L1), Cd38, and Cd52 was found in all Met2 cells. Cd274 is a well-established immune checkpoint molecule involved in immune escape, while less-studied molecules such as Cd38, Cd52, and CD200 may also contribute to immune suppression and potentially play a more significant role in resistance to PD-L1 inhibition. Overexpression of CD38 in the tumor microenvironment (TME) fosters immune suppression by reducing cytotoxic T-cell activity (28). CD52, found in higher levels in metastatic breast cancer cells [both triple-negative breast cancer (TNBC) and non-TNBC], suppresses T-cell immunity by binding to the inhibitory ligand SIGLEC10, suggesting the potential for CD52-targeting therapies like alemtuzumab to reactivate T-cell immunity in metastatic thyroid or breast cancer (29, 30). The CD200–CD200R signaling axis, involved in immune suppression, plays a key role in the survival and spread of various cancers (31). Given the overexpression of these immune checkpoint molecules in metastatic thyroid cancer cells, they likely contribute to immune evasion during metastasis (30, 32, 33). Additionally, CD68, a pan-macrophage marker, is often expressed by metastatic tumor cells to escape macrophage-mediated phagocytosis and the cytotoxic effects of CD8+ T cells. Indeed, elevated macrophage antigen expression in tumor tissue may signal a prometastatic state, correlating with poor prognosis (34, 35). Arginase (Arg1) is well-known for regulating cancer cell immune escape within the TME (36), and Fcer1g is a key gene involved in cancer immune infiltration (37). Collectively, these genes likely cooperate to enable metastatic tumor cells to survive in circulation and colonize distant organs. The present study identifies inflammation and immune checkpoint dysregulation as pivotal pathways in thyroid cancer metastasis.

A significant increase in mutational load was observed in patients with the seven-gene expression signature. High tumor mutational burden has been proposed as a potential biomarker for predicting immunotherapy response, as it may lead to the generation of immunogenic neoantigens in certain cancer types (38, 39). Two common measures of tumor mutational burden are the tumor aneuploidy score and the fraction of the genome containing single-nucleotide polymorphisms affected by copy number alterations (39). Given that thyroid cancer patients with the seven-gene expression signature exhibit both an increased aneuploidy score and high PD-L1 expression, this signature may have potential as a predictive biomarker for immunotherapy response.

Chromosomal instability is known to drive metastasis through the activation of a cytosolic DNA response (40). When genomic DNA spills into the cytosol, it triggers the cGAS (cyclic GMP-AMP synthase) and STING (Stimulator of interferon genes) pathways, which are key to sensing cytosolic DNA (41), and subsequently activate downstream noncanonical NF-κB signaling. Tumor cells with chromosomal instability often rely on the chronic activation of innate immune pathways to facilitate metastasis to distant organs (40). The major outcomes of chromosomal instability include ER stress and immune suppression (42). The enrichment of inflammation and innate immune pathways observed in the seven-gene expression signature further supports the involvement of chromosomal instability in thyroid cancer metastasis.

The limitation of this study is that the seven-gene expression signature was validated using a single TCGA dataset. Further validations in larger datasets and more diverse datasets would strengthen the study.

In summary, we have identified a seven-gene expression signature associated with poor prognosis and chromosomal instability. These genes may serve as valuable biomarkers for risk stratification of distant metastasis and/or high-risk lymph node metastasis, potentially aiding in decision-making for initial surgical recommendations.

## Data Availability

The datasets presented in this study can be found in online repositories. The names of the repository/repositories and accession number(s) can be found in the article/**Supplementary Material.** All data are included in the manuscript and supplementary data including the Gene Expression Omnibus (GEO) repository (GSE284225).
