# Supplementary material for: Identification of novel prognostic biomarkers for thyroid cancer by integrated transcriptome analysis of metastasis-associated genes
Source: Front Oncol. 2025 May 19;15:1536270. doi: 10.3389/fonc.2025.1536270 (PMC12127207; doi:10.3389/fonc.2025.1536270)
Supplement: Supplementary file 1 [file Table1.docx]

**Supplementary Table 1. 130 DEGs present in all Met2 cell lines**

| **Gene ID** | **Gene Symbol** | **FTC-Met2** | **PDTC-Met2** | **PTC-Met2** | **ATC-Met2** |
| --- | --- | --- | --- | --- | --- |
| 72925 | MARCHF1 | 4 | 4.087463 | 6.629357 | 5.4918531 |
| 11421 | Ace | 4.445818619 | 6.163230349 | 2.321928095 | 7.592457037 |
| 13733 | Adgre1 | 3.584962501 | 8.224001674 | 11.02097994 | 10.02928723 |
| 107747 | Aldh1l1 | 8.596189756 | 3.680857928 | 3.378511623 | 7.294620749 |
| 11690 | Alox5ap | 5.285402219 | 4.834576391 | 5.994579724 | 12.09242682 |
| 105827 | Amigo2 | 3.906890596 | 5.321928095 | 8.044394119 | 4.087462841 |
| 77531 | Anks1b | 2.475733431 | 5.523561956 | 3.357552005 | 3.672425342 |
| 75761 | Apol7a | 3.421463768 | 2.5360529 | 4.754887502 | 3.321928095 |
| 11846 | Arg1 | 5.392317423 | 11.55650605 | 11.2179577 | 15.65079933 |
| 216445 | Arhgap9 | 5.209453366 | 6.64385619 | 6.725001003 | 8.056637715 |
| 219144 | Arl11 | 2.30580843 | 3.902512155 | 8.670656249 | 5.317593505 |
| 74008 | Arsg | 2.099535674 | 5.209453366 | 4.280107919 | 5.345774837 |
| 57344 | As3mt | 7.5025 | 2.157541 | 2.448758 | 2.602036 |
| 53313 | Atp2a3 | 2.584963 | 2.61471 | 4.837943 | 3.5037122 |
| 12259 | C1qa | 4.64385619 | 9.231221181 | 11.57695666 | 12.49660415 |
| 12351 | Car4 | 4.321928095 | 10.6926155 | 10.52552081 | 8.511752654 |
| 108723 | Card11 | 2.584962501 | 4.459431619 | 7.312882955 | 5.491853096 |
| 12424 | Cck | 5.977279923 | 8.21916852 | 5.7642862 | 12.64069684 |
| 20302 | Ccl3 | 5.196397213 | 10.43879185 | 13.10901442 | 12.06676193 |
| 20305 | Ccl6 | 4.66106548 | 12.2822197 | 14.45545566 | 11.40118722 |
| 17470 | Cd200 | 4.247928 | 2.570316 | 8.573647 | 3.5676845 |
| 60533 | Cd274 | 2.622437206 | 4.392317423 | 5.080373416 | 6.453956489 |
| 12494 | Cd38 | 7.098032 | 3.584963 | 3.494765 | 3.3719688 |
| 23833 | Cd52 | 5.700439718 | 7.714245518 | 12.10394343 | 12.76155123 |
| 12514 | Cd68 | 3.923689 | 3.925921 | 4.385778 | 3.2789349 |
| 17064 | Cd93 | 5.087462841 | 2 | 4.353636955 | 5.260235772 |
| 56620 | Clec4n | 3.070389328 | 11.68606275 | 13.83249448 | 13.46454575 |
| 170571 | Cntnap4 | 5.087462841 | 4.247927513 | 4.584962501 | 8.523561956 |
| 12835 | Col6a3 | 9.285402219 | 3.499146534 | 11.37123213 | 6.95419631 |
| 12903 | Crabp1 | 2.273349 | 4.640282 | 5.52803 | 2.0033357 |
| 12978 | Csf1r | 4.087462841 | 2.350497247 | 8.543805176 | 6.261423356 |
| 12983 | Csf2rb | 2.263034406 | 4.341219433 | 11.56938096 | 10.68270204 |
| 13040 | Ctss | 2.142957954 | 10.90162116 | 12.90388185 | 12.87670878 |
| 13058 | Cybb | 4.700439718 | 7.21916852 | 10.07146236 | 10.36632221 |
| 72318 | Cyth4 | 7.392317 | 2.27288 | 7.222622 | 3.377765 |
| 227929 | Cytip | 4.311586151 | 5.357552005 | 6.392317423 | 7.276124405 |
| 13586 | Ear1 | 5.491853096 | 10.07146236 | 12.15323534 | 11.7232343 |
| 503845 | Ear12 | 6.942514505 | 10.72109919 | 12.60894781 | 12.27699674 |
| 13587 | Ear2 | 5.672425342 | 10.2467406 | 11.62067804 | 10.30947635 |
| 66892 | Eif4e3 | 3.44625623 | 2.20469042 | 5.247927513 | 3.235216462 |
| 13982 | Esr1 | 4.954196 | 2.115477 | 2.13245 | 2.3692338 |
| 14127 | Fcer1g | 7.90000421 | 5.064718273 | 9.735146536 | 13.74336181 |
| 246256 | Fcgr4 | 4.523561956 | 5.562242424 | 9.968666793 | 9.977279923 |
| 14182 | Fgfr1 | 3.72377697 | 2.20650938 | 4.237669608 | 2.573841461 |
| 14652 | Glp1r | 7.857980995 | 5.709658248 | 5.523561956 | 8.409390936 |
| 217122 | Gm11545 | 4.247927513 | 5.087462841 | 4.539158811 | 7.62935662 |
| 93695 | Gpnmb | 4.523561956 | 12.12734954 | 13.42232762 | 10.70090649 |
| 106512 | Gpsm3 | 2.791099031 | 2.797484678 | 6.627821838 | 4.821880254 |
| 15896 | Icam2 | 4.523562 | 5.643856 | 6.108524 | 5.6438562 |
| 68713 | Ifitm1 | 6.794415866 | 3.855556767 | 5.682278733 | 9.923327485 |
| 16012 | Igfbp6 | 7.111464155 | 6.212574646 | 7.982845454 | 3.242428034 |
| 16170 | Il16 | 3.169925001 | 5.357552005 | 9.95419631 | 6.554588852 |
| 16181 | Il1rn | 5.129283017 | 3 | 4.320223117 | 5.689868588 |
| 16186 | Il2rg | 7.95419631 | 3.215874587 | 10.22400167 | 5.828990836 |
| 16331 | Inpp5d | 4.087462841 | 3.491853096 | 3.275634443 | 9.328674927 |
| 27356 | Insl6 | 2.038664 | 2.845829 | 2.471923 | 2.0951572 |
| 15900 | Irf8 | 4.481557281 | 2.396475048 | 4.240746339 | 9.418455019 |
| 16414 | Itgb2 | 2.102361718 | 6.034423831 | 5.863118194 | 5.551758848 |
| 223272 | Itgbl1 | 5.106915204 | 4.156504486 | 8.871135184 | 9.194756854 |
| 16792 | Laptm5 | 8.900866808 | 11.28915435 | 11.55090708 | 13.23187117 |
| 77889 | Lbh | 2.144920995 | 2.385493224 | 3.066790087 | 3.327173397 |
| 18826 | Lcp1 | 4.95419631 | 8.199672345 | 9.262094845 | 12.87152026 |
| 14728 | Lilrb4a | 6.426264755 | 4.487265827 | 4.84434913 | 6.355859296 |
| 107321 | Lpxn | 3.879145605 | 6.807354922 | 4.745954377 | 6.925124944 |
| 74511 | Lrrc17 | 5.491853096 | 6.820178962 | 4.807354922 | 3.584962501 |
| 17105 | Lyz2 | 3.144771524 | 15.92942024 | 12.28517207 | 18.38606035 |
| 74490 | Mamstr | 5.781359714 | 4.283383051 | 3.938599455 | 4.857980995 |
| 17167 | Marco | 4.169925001 | 11.91251514 | 9.991135289 | 4.599912842 |
| 68279 | Mcoln2 | 2.05626822 | 3.700439718 | 5.614709844 | 5.832890014 |
| 17289 | Mertk | 4.372554168 | 3.584962501 | 4.285402219 | 2.159198595 |
| 17381 | Mmp12 | 4.68182404 | 4.851749041 | 9.917869303 | 13.25841881 |
| 68774 | Ms4a6d | 4.459431619 | 7.971543554 | 10.74399286 | 10.98726401 |
| 17916 | Myo1f | 2.807354922 | 7.906890596 | 10.19967234 | 10.49785184 |
| 17969 | Ncf1 | 2.807354922 | 7.554588852 | 10.07012094 | 9.063395081 |
| 105855 | Nckap1 | 5.426264755 | 4.437405312 | 9.638435914 | 10.3858624 |
| 407790 | Ndufa4l2 | 3.911357147 | 5.882643049 | 5.614709844 | 10.6183855 |
| 434341 | Nlrc5 | 5.857981 | 4.087463 | 2.690316 | 4.2976805 |
| 216799 | Nlrp3 | 4.392317423 | 5.058893689 | 9.027905997 | 8.918863237 |
| 107607 | Nod1 | 5.604862058 | 2.512166675 | 7.22881869 | 4.541373232 |
| 224109 | Nrros | 3 | 7.599912842 | 8.087462841 | 5.765126687 |
| 140795 | P2RY14 | 4 | 2.095157 | 6.129283 | 7.0980321 |
| 14726 | Pdpn | 2.187255581 | 7 | 5.882319139 | 11.94727165 |
| 218194 | Phactr1 | 2.95419631 | 3.027480736 | 2.807354922 | 4.357552005 |
| 104759 | Pld4 | 4.307428525 | 2.839959587 | 11.3858624 | 9.205060111 |
| 235527 | Plscr4 | 3.150941898 | 2.538419915 | 6.539158811 | 4.64385619 |
| 72324 | Plxdc1 | 7.357552005 | 4.619608644 | 6.548436625 | 4.14974712 |
| 243743 | Plxna4 | 3 | 3.169925 | 5.539159 | 2.6114347 |
| 19222 | Ptgir | 3.459431619 | 3.155278225 | 5.683696454 | 7.044394119 |
| 19731 | Rgl1 | 2.318698007 | 2.667855509 | 4.061296179 | 5.800703842 |
| 380713 | Scarf1 | 3.169925001 | 5.375039431 | 7 | 5.392317423 |
| 107303348 | SETDB2-PHF11C | 6.459432 | 3.70044 | 5.209453 | 2.3692338 |
| 58234 | Shank3 | 2.271596419 | 2.717856771 | 6.06608919 | 4.022367813 |
| 20612 | Siglec1 | 2.807354922 | 7.118941073 | 10.2632692 | 8.965784285 |
| 620235 | Siglec15 | 2.38466385 | 3.654864514 | 4.56193706 | 2.339850003 |
| 65221 | Slc15a3 | 4.058893689 | 2.450056088 | 11.85018684 | 11.71467483 |
| 13602 | Sparcl1 | 4.431845787 | 4.392317423 | 10.41785251 | 5.614709844 |
| 21391 | Tbxas1 | 5.357552005 | 7.894817763 | 9.224001674 | 10.32755264 |
| 23965 | Tenm3 | 7.163901214 | 3 | 5.807354922 | 5.569855608 |
| 23966 | Tenm4 | 3.459431619 | 5 | 2.807354922 | 3.218423519 |
| 24088 | TLR2 | 2.413521 | 3.077243 | 4.976364 | 3.3405297 |
| 21926 | Tnf | 4.169925001 | 3.574908836 | 8.977279923 | 3.972692654 |
| 22041 | Trf | 5.906890596 | 5.071587082 | 11.58824615 | 13.98708633 |
| 27027 | Tspan32 | 4.584963 | 4.392317 | 3.142958 | 2.5380959 |
| 22376 | Was | 2.632268215 | 3.886132035 | 9.177419538 | 6.037089319 |
| 100034251 | Wfdc17 | 8.164906927 | 8.583082768 | 14.81217731 | 11.87651695 |
| 74328 | 1700047E10Rik | -3.10433666 | -2.511819645 | -2.203807686 | -2.838381818 |
| 71874 | 2310007B03Rik or Mab21L4 | -4.350497247 | -8.768184325 | -10.72536626 | -12.34346334 |
| 11647 | Alpl | -5.93074 | -7.09346 | -5.006426 | -5.149747 |
| 57278 | Bcam | -2.295092622 | -4.565257091 | -7.58954712 | -6.451623252 |
| 67445 | C1qtnf4 | -5.426264755 | -6.456149035 | -6.129283017 | -5.028408415 |
| 66371 | Chmp4c | -2.561878888 | -5.459431619 | -5.390407833 | -5.501439145 |
| 12804 | Cntfr | -5.584962501 | -6.108524457 | -5.934673752 | -3.361456459 |
| 12829 | Col4a4 | -2.550197083 | -3.700439718 | -5.033423002 | -2.892570924 |
| 13106 | Cyp2e1 | -3.96212482 | -5.842350343 | -7.328674927 | -2.469726414 |
| 14073 | Faah | -3.584962501 | -4.459431619 | -6.894817763 | -5.205548911 |
| 64339 | Fndc4 | -4.340368678 | -6.222112368 | -2.782233809 | -2.496874538 |
| 319167 | Hist1h2ag | -8.654636029 | -4.312239804 | -2.342035668 | -7.434628228 |
| 17388 | Mmp15 | -5.285402219 | -6.209453366 | -5.194756854 | -7.436711542 |
| 74337 | Palm3 | -4.90689 | -5.58496 | -4.877744 | -5.533979 |
| 50873 | Park2 | -11.77941158 | -3.169003667 | -4.921570491 | -8.161111227 |
| 18510 | Pax8 | -3.321928095 | -4.321928095 | -7.577428828 | -4.024946357 |
| 215789 | Phactr2 | -4.75489 | -3 | -4.169925 | -2.091922 |
| 19208 | Ptcra | -4.182203331 | -2.574470127 | -4.135159583 | -2.7589919 |
| 19268 | Ptprf | -2.775473414 | -3.147643554 | -4.840761007 | -5.06463135 |
| 72433 | Rab38 | -5.459431619 | -2.347923303 | -7.082149041 | -4.380821784 |
| 234214 | Sorbs2 | -5.101538026 | -5.129283017 | -4.584962501 | -8.586214297 |
| 20666 | Sox11 | -6.189824559 | -3.029747343 | -4.584962501 | -7.108524457 |
| 66260 | Tmem54 | -4.614709844 | -4.26497221 | -11.00457162 | -12.31061278 |
| 21912 | Tspan7 | -8.988684687 | -9.299208018 | -2.523754946 | -5.849162407 |
| 665976 | Vmn2r-ps129 | -3.641344971 | -2.007494537 | -2.648288436 | -3.469114172 |

*Log2FoldChange
