# Supplementary material for: Identification of novel prognostic biomarkers for thyroid cancer by integrated transcriptome analysis of metastasis-associated genes
Source: Front Oncol. 2025 May 19;15:1536270. doi: 10.3389/fonc.2025.1536270 (PMC12127207; doi:10.3389/fonc.2025.1536270)
Supplement: Supplementary file 2 [file Table2.docx]

**Supplementary Table 2. Hub genes in thyroid cancer metastasis**

|  | **Gene symbol**  **(degree of node)** | **Description** | **Biological role in cancer** | **Reference** |
| --- | --- | --- | --- | --- |
| **1** | **TNF (42)** | Tumor Necrosis Factor | A multifunctional proinflammatory cytokine. Involved in the regulation of a wide spectrum of biological processes including cell proliferation, differentiation, apoptosis, lipid metabolism, and coagulation. High TNF levels impede tumor growth and low levels would promote cancer development and progression | **(**[**1**](#_ENREF_1)**)** |
| **2** | **CD68 (34)** | CD68 Molecule |  |  |
| **3** | **ITGB2 (34)** | Integrin Subunit Beta 2 | Heterodimeric leukocyte adhesion molecule essential for adhesion, trafficking and T cell effector function. Expression in cancer cells promotes invasion and metastasis in a manner mimicking leukocytes via YAP1, a downstream nuclear effector of the Hippo signaling pathway and PRDM4 | **(**[**2**](#_ENREF_2)**)** |
| **4** | **CSF1R (32)** | Colony Stimulating Factor 1 Receptor | Intratumor CSF-1/CSF-1R signaling is known to be overexpressed in many tumor types and associated with poor prognosis. Its activation causes recruitment of TAMs and development of pro-tumor inflammatory environment, leading to tumor growth and metastasis | **(**[**3**](#_ENREF_3)**,**[**4**](#_ENREF_4)**)** |
| **5** | **FCGR4 (FCGR3A) (30)** | Fc receptor, IgG, low affinity IV, closely related to human FCGR3A (Fc gamma RIIIA , 60% amino acid identity) | Enables IgE receptor activity and IgG receptor activity. Involved in neutrophil activation and tumor immunity. High expression of FCGR3A was associated with poor prognosis for patients with prostate cancer | **(**[**5**](#_ENREF_5)**,**[**6**](#_ENREF_6)**)** |
| **6** | **FCER1G (28)** | Fc Epsilon Receptor Ig | A key molecule involved in allergic inflammatory reactions. Up-regulated in most tumour and closely related to tumour microenvironment and tumour immunity. High expression of FCER1G in ccRCC is closely related to TAMs infiltration and suppression of T cell activation and proliferation. FCER1G-associated partners were enriched in pathways associated with neutrophils activation | **(**[**7**](#_ENREF_7)**)** |
| **7** | **TLR2 (27)** | Toll Like Receptor 2 |  |  |
| **8** | **IRF8 (24)** | Interferon Regulatory Factor 8 | A transcription factor of the interferon regulatory factor family and functions as tumor suppressor | **(**[**8**](#_ENREF_8)**)** |
| **9** | **CYBB (24)** | Cytochrome B-245 Beta Chain | A primary component of the microbicidal oxidase system of phagocytes that generates superoxide or reactive oxygen species (ROS). Genetic depletion of any of the NOX2 subunits Cyba, Cybb, Ncf1, Ncf2 and Ncf4 reduced the formation of lung metastases | **(**[**9**](#_ENREF_9)**)** |
| **10** | **C1QA (23)** | Complement C1q A Chain | C1q is the activator of the classical complement pathway. C1q acts in the tumour microenvironment as a cancer-promoting factor unrelated to complement activation | **(**[**10**](#_ENREF_10)**,**[**11**](#_ENREF_11)**)** |
| **11** | **CD274 (22)** | Programmed Cell Death 1 Ligand 1 | A ligand that binds with the receptor PD1, commonly found on T-cells, and acts to block T-cell activation | **(**[**12**](#_ENREF_12)**)** |
| **12** | **LAPTM5 (22)** | Lysosomal Protein Transmembrane 5 | It promotes lung-specific metastasis in renal cancer. LAPTM5 overexpression in lung metastases is a common phenomenon in multiple cancer types and a potential therapeutic target for cancers with lung metastasis | **(**[**13**](#_ENREF_13)**)** |
| **13** | **CTSS (20)** | Cathepsin S | A lysosomal cysteine proteinase capable of degrading the extracellular matrix and promoting cell metastasis | **(**[**14**](#_ENREF_14)**)** |
| **14** | **CCL3 (19)** | C-C Motif Chemokine Ligand 3 | CCL3–CCR5 axis contributes to progression of esophageal squamous cell carcinoma by promoting cell migration and invasion via Akt and ERK pathways | **(**[**15**](#_ENREF_15)**)** |
| **15** | **SIGLEC1 (17)** | Sialic Acid Binding Ig Like Lectin 1 | A lectin-like adhesion molecule that binds glycoconjugate ligands on cell surfaces in a sialic acid-dependent manner. Hypersialylation promotes tumor metastasis by enhancing immune evasion and stimulating tumor invasion and migration. | **(**[**16**](#_ENREF_16)**)** |
| **16** | **LYZ (16)** | Lysozyme | mRNA level of LYZ is an immune-related marker to predict the prognosis or response of these tumors to immune therapies | **(**[**17**](#_ENREF_17)**)** |
| **17** | **CD52 (16)** | CD52 Molecule | A glycoprotein anchored on the cell membrane by a glycosylphosphatidylinositol (GPI). Soluble CD52 binds to sialic acid-binding immunoglobulin-like lectin-10 (Siglec10), a signal molecule of immunosuppression on the surface of T cells | **(**[**18**](#_ENREF_18)**,**[**19**](#_ENREF_19)**)** |
| **18** | **ARG1 (16)** | Arginase 1 | It catalyzes the hydrolysis of L-arginine to L-ornithine and urea. The depletion of L-arginine leads to immune escape of cancer cells | **(**[**20**](#_ENREF_20)**)** |
| **19** | **NLRP3 (15)** | NLR Family Pyrin Domain Containing 3 | The NLRP3 inflammasome is a multimeric cytosolic protein complex and functions as an upstream activator of NF-kappaB signaling. It plays a role in the regulation of inflammation, immune response, and apoptosis. It is involved in the activation of caspase-1, leading to secretion of inflammatory cytokines IL1B and IL18 and inflammatory cell death, pyroptosis | **(**[**21**](#_ENREF_21)**)** |
| **20** | **CD38 (13)** | ADP-Ribosyl Cyclase 1 | An enzyme involved in both the hydrolysis and biosynthesis of cyclic ADP-ribose (cADPR). It is a major mechanism of acquired resistance to PD-1/PD-L1 blockade, causing CD8+ T-cell suppression | **(**[**22**](#_ENREF_22)**)** |
| **21** | **NCF1 (12)** | Neutrophil Cytosolic Factor 1 | A cytosolic component of the NADPH oxidase 2 (NOX2) complex required for the production of reactive oxygen species (ROS), which promotes metastatic colonization | **(**[**23**](#_ENREF_23)**)** |
| **22** | **IL2RG (11)** | Interleukin 2 Receptor Subunit Gamma | An important cytokine receptor sub-unit that is common to at least six different interleukin receptors: IL-2, IL-4, IL-7, IL-9, IL-15 and interleukin-21 receptor. Its overexpression mediates cancer cell growth through the JAK/Stat pathway | **(**[**24**](#_ENREF_24)**)** |
| **23** | **MERTK (11)** | MER Proto-Oncogene, Tyrosine Kinase | A member of the TYRO3, AXL, and MERTK (TAM) family of receptor tyrosine kinases. It activates multiple signaling pathways (MAPK, PI3K/AKT, JAK/STAT, and PD-1/PD-L1) in many types of cancer to promote immune tolerance, tumor progression and metastasis, and drug resistance | **(**[**25**](#_ENREF_25)**)** |
| **24** | **CD93 (11)** | CD93 Molecule | A receptor for insulin-like growth factor binding protein 7 and interacts with and promotes the activation of integrin β1. It maintains endothelial barrier function and limits metastatic dissemination | **(**[**26**](#_ENREF_26)**)** |
| **25** | **NCKAP1L (11)** | NCK Associated Protein 1 Like | A hematopoietic lineage-specific regulator of the actin cytoskeleton. Nckap1l-deficient mice have anomalies in lymphocyte development, phagocytosis, and neutrophil migration. Its role in cancer metastasis is not clear, but its paralog regulates metastasis and is a novel prognostic marker for colorectal cancer | **(**[**27**](#_ENREF_27)**)** |
| **26** | **MYO1F (11)** | Myosin IF | A family of non-processive molecular motors attached to actin filaments; the interaction between these proteins is involved in tumor cell migration | **(**[**28**](#_ENREF_28)**)** |
| **27** | **LCP1 (11)** | Lymphocyte Cytosolic Protein 1 | An actin-binding protein involved in adhesion and actin assembly, which has been shown to contribute significantly to the invasiveness of tumors | **(**[**29**](#_ENREF_29)**,**[**30**](#_ENREF_30)**)** |
| **28** | **ARHGAP9 (10)** | Rho GTPase Activating Protein 9 | A member of the Rho-GAP family of GTPase activating proteins which plays an important role in cell adhesion and migration. Its overexpression was associated with poor OS in AML patients | **(**[**31**](#_ENREF_31)**)** |
| **29** | **WAS (10)** | WASP Actin Nucleation Promoting Factor | Wiskott-Aldrich Syndrome protein (WASp) is an actin nucleation-promoting factor and is a key regulator of actin polymerization involved in cancer metastasis. | **(**[**32**](#_ENREF_32)**,**[**33**](#_ENREF_33)**)** |
| **30** | **MS4A6D (MS4A6A) (10)** | Membrane Spanning 4-Domains A6A | A coreceptor of MHC class II antigen (MHC-II) that promotes macrophages-derived inflammation | **(**[**34**](#_ENREF_34)**,**[**35**](#_ENREF_35)**)** |
| **31** | **CSF2RB (10)** | Colony Stimulating Factor 2 Receptor Subunit Beta | CSF2RB (CD131) is the common subunit of the type I cytokine receptors for granulocyte-macrophage colony-stimulating factor (GM-CSF), interleukin (IL)-3 and IL-5. FOXP3+ regulatory T cells (Tregs) highly overexpress CSF2RB. Involved in pro-inflammatory TAM activation in brain metastasis | **(**[**36**](#_ENREF_36)**)** |
| **32** | **IL1RN (10)** | Interleukin 1 Receptor Antagonist | A potent anti-inflammatory molecule that modulates the biological activity of the proinflammatory cytokine, interleukin-1. | **(**[**37**](#_ENREF_37)**)** |

**Supplementary References**

1. Montfort A, Colacios C, Levade T, Andrieu-Abadie N, Meyer N, Ségui B. The TNF Paradox in Cancer Progression and Immunotherapy. *Frontiers in immunology*. 2019;10:1818.

2. Liu H, Dai X, Cao X, Yan H, Ji X, Zhang H, Shen S, Si Y, Zhang H, Chen J, Li L, Zhao JC, Yu J, Feng XH, Zhao B. PRDM4 mediates YAP-induced cell invasion by activating leukocyte-specific integrin β2 expression. *EMBO reports*. 2018;19(6).

3. Patsialou A, Wyckoff J, Wang Y, Goswami S, Stanley ER, Condeelis JS. Invasion of human breast cancer cells in vivo requires both paracrine and autocrine loops involving the colony-stimulating factor-1 receptor. *Cancer research*. 2009;69(24):9498-9506.

4. Ryder M, Gild M, Hohl TM, Pamer E, Knauf J, Ghossein R, Joyce JA, Fagin JA. Genetic and pharmacological targeting of CSF-1/CSF-1R inhibits tumor-associated macrophages and impairs BRAF-induced thyroid cancer progression. *PloS one*. 2013;8(1):e54302.

5. Zha Z, Hong Y, Tang Z, Du Q, Wang Y, Yang S, Wu Y, Tan H, Jiang F, Zhong W. FCGR3A: A new biomarker with potential prognostic value for prostate cancer. *Frontiers in oncology*. 2022;12:1014888.

6. Li L, Huang Z, Du K, Liu X, Li C, Wang D, Zhang Y, Wang C, Li J. Integrative Pan-Cancer Analysis Confirmed that FCGR3A is a Candidate Biomarker Associated With Tumor Immunity. *Frontiers in pharmacology*. 2022;13:900699.

7. Yang R, Chen Z, Liang L, Ao S, Zhang J, Chang Z, Wang Z, Zhou Y, Duan X, Deng T. Fc Fragment of IgE Receptor Ig (FCER1G) acts as a key gene involved in cancer immune infiltration and tumour microenvironment. *Immunology*. 2023;168(2):302-319.

8. Yang D, Thangaraju M, Greeneltch K, Browning DD, Schoenlein PV, Tamura T, Ozato K, Ganapathy V, Abrams SI, Liu K. Repression of IFN regulatory factor 8 by DNA methylation is a molecular determinant of apoptotic resistance and metastatic phenotype in metastatic tumor cells. *Cancer research*. 2007;67(7):3301-3309.

9. Martner A, Aydin E, Hellstrand K. NOX2 in autoimmunity, tumor growth and metastasis. *The Journal of pathology*. 2019;247(2):151-154.

10. Bulla R, Tripodo C, Rami D, Ling GS, Agostinis C, Guarnotta C, Zorzet S, Durigutto P, Botto M, Tedesco F. C1q acts in the tumour microenvironment as a cancer-promoting factor independently of complement activation. *Nature communications*. 2016;7:10346.

11. Roumenina LT, Daugan MV, Noé R, Petitprez F, Vano YA, Sanchez-Salas R, Becht E, Meilleroux J, Clec'h BL, Giraldo NA, Merle NS, Sun CM, Verkarre V, Validire P, Selves J, Lacroix L, Delfour O, Vandenberghe I, Thuilliez C, Keddani S, Sakhi IB, Barret E, Ferré P, Corvaïa N, Passioukov A, Chetaille E, Botto M, de Reynies A, Oudard SM, Mejean A, Cathelineau X, Sautès-Fridman C, Fridman WH. Tumor Cells Hijack Macrophage-Produced Complement C1q to Promote Tumor Growth. *Cancer immunology research*. 2019;7(7):1091-1105.

12. Gong J, Chehrazi-Raffle A, Reddi S, Salgia R. Development of PD-1 and PD-L1 inhibitors as a form of cancer immunotherapy: a comprehensive review of registration trials and future considerations. *Journal for immunotherapy of cancer*. 2018;6(1):8.

13. Jiang B, Zhao X, Chen W, Diao W, Ding M, Qin H, Li B, Cao W, Chen W, Fu Y, He K, Gao J, Chen M, Lin T, Deng Y, Yan C, Guo H. Lysosomal protein transmembrane 5 promotes lung-specific metastasis by regulating BMPR1A lysosomal degradation. *Nature communications*. 2022;13(1):4141.

14. Hsin MC, Hsieh YH, Wang PH, Ko JL, Hsin IL, Yang SF. Hispolon suppresses metastasis via autophagic degradation of cathepsin S in cervical cancer cells. *Cell death & disease*. 2017;8(10):e3089.

15. Kodama T, Koma YI, Arai N, Kido A, Urakawa N, Nishio M, Shigeoka M, Yokozaki H. CCL3-CCR5 axis contributes to progression of esophageal squamous cell carcinoma by promoting cell migration and invasion via Akt and ERK pathways. *Laboratory investigation; a journal of technical methods and pathology*. 2020;100(9):1140-1157.

16. Dobie C, Skropeta D. Insights into the role of sialylation in cancer progression and metastasis. *British journal of cancer*. 2021;124(1):76-90.

17. Gu Z, Wang L, Dong Q, Xu K, Ye J, Shao X, Yang S, Lu C, Chang C, Hou Y, Zhai Y, Wang X, He F, Sun A. Aberrant LYZ expression in tumor cells serves as the potential biomarker and target for HCC and promotes tumor progression via csGRP78. *Proceedings of the National Academy of Sciences of the United States of America*. 2023;120(29):e2215744120.

18. Bandala-Sanchez E, N GB, Goddard-Borger ED, Ngui K, Naselli G, Stone NL, Neale AM, Pearce LA, Wardak A, Czabotar P, Haselhorst T, Maggioni A, Hartley-Tassell LA, Adams TE, Harrison LC. CD52 glycan binds the proinflammatory B box of HMGB1 to engage the Siglec-10 receptor and suppress human T cell function. *Proceedings of the National Academy of Sciences of the United States of America*. 2018;115(30):7783-7788.

19. Xu K, Wang R, Xie H, Hu L, Wang C, Xu J, Zhu C, Liu Y, Gao F, Li X, Wang C, Huang J, Zhou W, Zhou G, Shu Y, Guan X. Single-cell RNA sequencing reveals cell heterogeneity and transcriptome profile of breast cancer lymph node metastasis. *Oncogenesis*. 2021;10(10):66.

20. Niu F, Yu Y, Li Z, Ren Y, Li Z, Ye Q, Liu P, Ji C, Qian L, Xiong Y. Arginase: An emerging and promising therapeutic target for cancer treatment. *Biomedicine & pharmacotherapy = Biomedecine & pharmacotherapie*. 2022;149:112840.

21. Sharma BR, Kanneganti TD. NLRP3 inflammasome in cancer and metabolic diseases. *Nature immunology*. 2021;22(5):550-559.

22. Chen L, Diao L, Yang Y, Yi X, Rodriguez BL, Li Y, Villalobos PA, Cascone T, Liu X, Tan L, Lorenzi PL, Huang A, Zhao Q, Peng D, Fradette JJ, Peng DH, Ungewiss C, Roybal J, Tong P, Oba J, Skoulidis F, Peng W, Carter BW, Gay CM, Fan Y, Class CA, Zhu J, Rodriguez-Canales J, Kawakami M, Byers LA, Woodman SE, Papadimitrakopoulou VA, Dmitrovsky E, Wang J, Ullrich SE, Wistuba, II, Heymach JV, Qin FX, Gibbons DL. CD38-Mediated Immunosuppression as a Mechanism of Tumor Cell Escape from PD-1/PD-L1 Blockade. *Cancer discovery*. 2018;8(9):1156-1175.

23. Zhong J, Li Q, Luo H, Holmdahl R. Neutrophil-derived reactive oxygen species promote tumor colonization. *Communications biology*. 2021;4(1):865.

24. Ayars M, O'Sullivan E, Macgregor-Das A, Shindo K, Kim H, Borges M, Yu J, Hruban RH, Goggins M. IL2RG, identified as overexpressed by RNA-seq profiling of pancreatic intraepithelial neoplasia, mediates pancreatic cancer growth. *Oncotarget*. 2017;8(48):83370-83383.

25. Lahey KC, Gadiyar V, Hill A, Desind S, Wang Z, Davra V, Patel R, Zaman A, Calianese D, Birge RB. Mertk: An emerging target in cancer biology and immuno-oncology. *International review of cell and molecular biology*. 2022;368:35-59.

26. Vemuri K, de Alves Pereira B, Fuenzalida P, Subashi Y, Barbera S, van Hooren L, Hedlund M, Pontén F, Lindskog C, Olsson AK, Lugano R, Dimberg A. CD93 maintains endothelial barrier function and limits metastatic dissemination. *JCI insight*. 2024;9(7).

27. Kwon MR, Lee JH, Park J, Park SS, Ju EJ, Ko EJ, Shin SH, Son GW, Lee HW, Kim YJ, Song SY, Jeong SY, Choi EK. NCK-associated protein 1 regulates metastasis and is a novel prognostic marker for colorectal cancer. *Cell Death Discov*. 2023;9(1):7.

28. Diaz-Valencia JD, Estrada-Abreo LA, Rodríguez-Cruz L, Salgado-Aguayo AR, Patiño-López G. Class I Myosins, molecular motors involved in cell migration and cancer. *Cell adhesion & migration*. 2022;16(1):1-12.

29. Pan S, Wan M, Jin H, Ning R, Zhang J, Han X. LCP1 correlates with immune infiltration: a prognostic marker for triple-negative breast cancer. *BMC immunology*. 2024;25(1):42.

30. Ge X, Liu W, Zhao W, Feng S, Duan A, Ji C, Shen K, Liu W, Zhou J, Jiang D, Rong Y, Gong F, Wang J, Xu Z, Li X, Fan J, Wei Y, Bai J, Cai W. Exosomal Transfer of LCP1 Promotes Osteosarcoma Cell Tumorigenesis and Metastasis by Activating the JAK2/STAT3 Signaling Pathway. *Molecular therapy Nucleic acids*. 2020;21:900-915.

31. Han C, He S, Wang R, Gao X, Wang H, Qiao J, Meng X, Li Y, Yu L. The role of ARHGAP9: clinical implication and potential function in acute myeloid leukemia. *Journal of translational medicine*. 2021;19(1):65.

32. Lane J, Martin T, Weeks HP, Jiang WG. Structure and role of WASP and WAVE in Rho GTPase signalling in cancer. *Cancer genomics & proteomics*. 2014;11(3):155-165.

33. Biber G, Ben-Shmuel A, Noy E, Joseph N, Puthenveetil A, Reiss N, Levy O, Lazar I, Feiglin A, Ofran Y, Kedmi M, Avigdor A, Fried S, Barda-Saad M. Targeting the actin nucleation promoting factor WASp provides a therapeutic approach for hematopoietic malignancies. *Nature communications*. 2021;12(1):5581.

34. Chen Y, Li S, Huang X, Wang C, Pan Y, Xiang Q, Feng Z, Fei L, Wu Y, Ruan Z, An Y, Chen Y. Tetraspan MS4A6D is a coreceptor of MHC class II antigen (MHC-II) that promotes macrophages-derived inflammation. *Molecular immunology*. 2023;160:121-132.

35. Schulz M, Sevenich L. TAMs in Brain Metastasis: Molecular Signatures in Mouse and Man. *Frontiers in immunology*. 2021;12:716504.

36. Klemm F, Möckl A, Salamero-Boix A, Alekseeva T, Schäffer A, Schulz M, Niesel K, Maas RR, Groth M, Elie BT, Bowman RL, Hegi ME, Daniel RT, Zeiner PS, Zinke J, Harter PN, Plate KH, Joyce JA, Sevenich L. Compensatory CSF2-driven macrophage activation promotes adaptive resistance to CSF1R inhibition in breast-to-brain metastasis. *Nature cancer*. 2021;2(10):1086-1101.

37. Wu TC, Xu K, Martinek J, Young RR, Banchereau R, George J, Turner J, Kim KI, Zurawski S, Wang X, Blankenship D, Brookes HM, Marches F, Obermoser G, Lavecchio E, Levin MK, Bae S, Chung CH, Smith JL, Cepika AM, Oxley KL, Snipes GJ, Banchereau J, Pascual V, O'Shaughnessy J, Palucka AK. IL1 Receptor Antagonist Controls Transcriptional Signature of Inflammation in Patients with Metastatic Breast Cancer. *Cancer research*. 2018;78(18):5243-5258.
